# Regulation of resveratrol biosynthesis in grapevine: new approaches for disease resistance?

**DOI:** 10.1093/jxb/ery446

**Published:** 2019-01-07

**Authors:** Philippe Jeandet, Christophe Clément, Sylvain Cordelier

**Affiliations:** Research Unit, Induced Resistance and Plant Bioprotection, EA 4707, SFR Condorcet FR CNRS 3417, Faculty of Sciences, University of Reims Champagne-Ardenne, 51687 Reims Cedex 2, France

**Keywords:** Grapevine, phenylpropanoid biosynthesis, protein–protein interactions, regulation, resveratrol, secondary metabolism, VvMYB14, VvWRKY8

## Abstract

This article comments on:

**Jiang J, Xi H, Dai Z, Lecourieux F, Yuan L, Liu X, Patra B, Wei Y, Li S, Wang L.** 2019. VvWRKY8 negatively regulates VvSTS through direct interaction with VvMYB14 to balance resveratrol biosynthesis in grapevine. Journal of Experimental Botany 70, 715–729.

**Transcription factors are key components in the regulation of metabolic pathways underlying numerous plant functions. Jiang *et al.* (2019) showed that the WRKY8 transcription factor fine-tunes biosynthesis of the phytoalexin resveratrol in grapevine through negative regulation of the stilbene synthase gene. This paves the way for new approaches in our understanding of the regulation of phytoalexin biosynthesis in plants and, through this, improved phytoalexin production in engineered disease resistance.**

During their life cycle, plants face a large number of abiotic and biotic stresses including salt, cold, heat, drought, wounding, osmotic pressure, UV and pathogenic microorganisms. In order to cope, an intricate network has developed comprising stress-responsive signal transduction pathways and adaptive mechanisms involving both primary and secondary metabolism. Regulation of the plant transcriptome involved in these responses requires the action of an impressive predicted number of transcription factors (TFs), varying from 1500 to 1600 according to genome-wide identification analyses carried out in rice and Arabidopsis (Iida *et al.*, 2005; Xiong *et al.*, 2005; Agarwal *et al.*, 2011).

WRKY proteins have been recognized as one of the ten largest families of TFs in higher plants, though they are absent in animals (Llorca *et al.*, 2014). They are so called because of their characteristic DNA-binding domain of around 60 amino acids which contains, either once or twice, the quasi-invariant WRKYGQK amino acid sequence at the N-terminus and a zinc-finger structure at the C-terminus constituting the WRKY domain (Llorca *et al.*, 2014). Both the WRKYGQK motif and the zinc finger are necessary for the DNA binding of TFs. Specifically, the WRKY domain binds the W-box (TTGACC/T) motifs of the promoters of target genes to modulate their expression (Llorca *et al.*, 2014; Schluttenhofer and Yuan, 2015). In addition, the DNA sequences directly adjacent to the W-boxes have been shown to be important for the specific DNA-binding activity of the WRKY TFs (Ciolkowski *et al.*, 2008).

Most WRKY TFs are located in the nucleus though some studies have reported the cytosolic localization of the Arabidopsis AtWRKY40, which inhibits the expression of ABA-responsive genes (Shang *et al.*, 2010). TFs play a role as positive and negative regulators of major plant functions such as growth, development and senescence, defence, abiotic stresses and hormone signalling (Agarwal *et al.*, 2011). For example, AtWRKY6 positively influences the promoter activity of the senescence *SIRK* gene while AtWRKY44 (TTG2) controls trichome and seed coat development in Arabidopsis (Johnson *et al.*, 2002; Robatzek and Somssich, 2002). Importantly, WRKY TFs were also shown to be key regulators of plant secondary metabolism (Schluttenhofer and Yuan, 2015), including phytoalexin biosynthesis (Jiang *et al.*, 2019).

## WRKY TFs are key regulators of plant secondary metabolism

WRKY TFs intervene in numerous pathways of secondary metabolism relating to a wide array of biological functions in plants (Schluttenhofer and Yuan, 2015). They are also implicated in the biosynthesis of many metabolites of pharmaceutical significance. *Artemisia annua* AaWRKY1 positively regulates a cytochrome P450 monooxygenase in the biosynthetic route to the antimalarial drug artemisinin (Chen *et al.*, 2017), whereas the *Taxus chinensis* TcWRKY1 regulates a 10‐deacetylbaccatin III‐10 β‐*O*‐acetyl transferase, a critical enzyme in the biosynthesis of the anticancer drug taxol (Li *et al.*, 2013). TF expression is also linked to the biosynthesis of some phytoalexins, which are secondary metabolites of low molecular weight synthesized by plants as a response to both abiotic and biotic stresses and displaying antimicrobial activity (Jeandet *et al.*, 2014). For example, AtWRKY40/18, *Oryza sativa* OsWRKY45 and *Vitis vinifera* VvWRKY8 TFs regulate the production of, respectively, camalexin in the Cruciferae (Pandey *et al.*, 2010), momilactone, oryzalexin and phytocassane phytoalexins in the Poaceae (Akagi *et al.*, 2014) and the resveratrol stilbene phytoalexin in the Vitaceae (Jiang *et al.*, 2019). Although resveratrol has been the subject of a plethora of research (Jeandet *et al.*, 2018), the mechanisms regulating the biosynthesis of this compound are still poorly understood. In their continuing efforts to decipher the regulatory mechanisms underlying the biosynthesis of resveratrol in Vitaceae, Jiang *et al.* (2019) report on the characterization of a WRKY transcription factor, VvWRKY8; this negatively regulates stilbene synthase, which catalyses the final committed step in the resveratrol pathway.

## Regulation mechanisms of stilbene synthase in Vitaceae

Resveratrol is obtained from the universal phenylpropanoid pathway starting either from phenylalanine or tyrosine and leading through two or three steps to *para*-coumaroyl-CoA. *Para*-coumaroyl-CoA is then linked to three malonyl-CoA units to form resveratrol under the action of the plant polyketide synthase III, stilbene synthase (STS) (Jeandet *et al.*, 2013, 2014, 2018) (Box 1). A few studies have reported on the regulatory mechanisms of the phenylpropanoid pathway and downstream pathways (lignins and flavonoids) in grapevine (*Vitis vinifera*). For example, the VvWRKY2 and VvMYB5a TFs were shown to activate transcription of the cinnamate-4-hydroxylase gene (*VvC4H*), suggesting their possible role in lignification processes in connection or not with plant disease resistance (Guillaumie *et al.*, 2010) or in anthocyanin biosynthesis (Deluc *et al.*, 2006). VvWRKY26 positively regulates chalcone synthase, the key enzyme of the flavonoid pathway as well as flavonoid hydroxylases (F3’H and F3’, 5’H) acting as decorating enzymes on the flavonoid core structure in grapevine (Amato *et al.*, 2016).

**Box 1. Resveratrol biosynthesis in grapevine leaves: model of the transcriptional regulatory loop**

Resveratrol is produced through the action of STS by condensation of *para*-coumaroyl-CoA and three malonyl CoA units. UV-C stress leads to induction of the *VvMYB14* gene (1). Accumulation of VvMYB14, with the combinatorial effect of VvWRKY3, results in the up-regulation of the *VvSTS29* gene (Vannozzi *et al.*, 2018) (2). At high concentration, resveratrol (R) induces *VvWRKY8* expression (possibly through a putative nuclear receptor), negatively regulating VvMYB14 and thereby stopping *VsSTS* expression and decreasing resveratrol production (3). At low resveratrol concentration, VvWRKY8 is channelled towards degradation through the ubiquitin ligase proteasome pathway, allowing the end of VvMYB14 repression and a new induction of *VvSTS* genes thereby increasing resveratrol production (4). Molecules of the resveratrol biosynthesis pathway are shown in blue; enzymes involved are white (PAL, phenylalanine ammonia lyase; C4H, cinnamate-4-hydroxylase; 4CL, *p*-coumarate-CoA ligase; STS, stilbene synthase) (Jeandet *et al.*, 2018). The MYB TFs are shown as blue ellipses; WRKY TFs are orange ellipses. The darker colours and bold arrows highlight the different actors, with some involved in the regulatory loop described in Jiang *et al.* (2019).

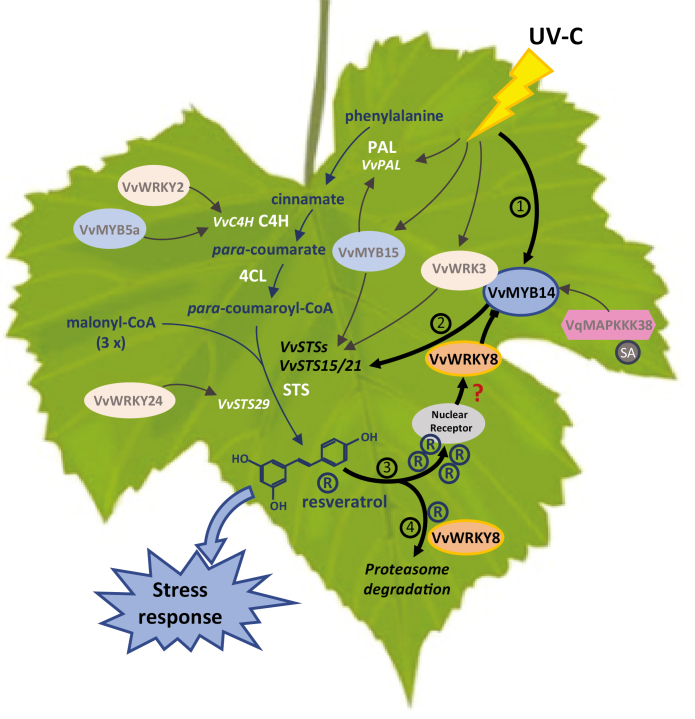

The first study implicating TFs in the regulation of STS in response to stress conditions identified two R2R3-MYB TFs, VvMYB14 and VvMYB15, for which the corresponding genes were strongly co-expressed with two *STS* genes, *VvSTS41*/*29* (Höll *et al.*, 2013). A further study from Corso *et al.* (2015), based on transcriptome comparison of two grapevine genotypes with different drought susceptibility, led to the identification of five additional WRKY TFs (*VvWRKY24/28/29/37/41*) that were co-expressed simultaneously with eight *VvSTS* transcripts (*VvSTS12*/*13*/*16*/*17*/*18*/*24*/*27*/*29*) in roots and leaves. Recently, the integrated Gene Coexpression Network (GCN) analysis of STS and TFs suggested that a great number of TFs belonging to various families such as WRKYs, MYBs and even ERFs can putatively contribute to STS regulation (Wong *et al.*, 2016; Vannozzi *et al.*, 2018). Indeed, three MYB TFs (*VvMYB13, VvMYB14* and *VvMYB15*) and four WRKY TFs (*VvWRKY3, VvWRKY24, VvWRKY43* and *VvWRKY53*) are co-expressed with *VvSTS* genes following biotic or abiotic stresses, raising the question of a possible role of these TFs in the regulation of *VvSTS* gene expression in *Vitis* spp. Moreover, VvWRKY24 induces the expression of the *VvSTS29* gene independently of VvMYB14 or VvMYB15 whereas VvWRKY3 and VvMYB14 have a combinatorial effect on the transcription of the *VvSTS29* gene (Vannozzi *et al.*, 2018) (Box 1). Genes of the MAPK pathway were also found to be involved in the activation of *STS* transcription in *V. quinquangularis* (Jiao *et al.*, 2017). A Raf-like *MAPKKK* gene, *VqMAPKKK38*, was indeed shown to positively regulate STS in grapevine leaves in combination with the salicylic acid hormone (SA), likely by induction of *VvMYB14* transcription. Overall, this highlights the intricate network underlying the regulation mechanisms of STS in grapevine. *VvSTS* genes can thus be regulated by the combinatorial action of MYB and WRKY transcription factors possibly in association with MAPKs (Box 1).

The research reported by Jiang *et al.* (2019) characterizes a negative regulator of resveratrol biosynthesis for the first time. It demonstrates a dosage-dependent inhibition of a *VvSTS*-inducing VvMYB14 TF by an N-terminus-mediated interaction with VvWRKY8 lacking transcriptional activity (Box 1). After UV-C exposure, the expression of *VvSTSs*, *VvWRKY8* and *VvMYB14* genes rose sharply in grapevine leaves. However, even if VvWRKY8 does not display any transcriptional activity in yeast, the transient *VvWRKY8* overexpression in grapevine leaves led to a down-regulation of *VvSTS15/21* and *VvMYB14* gene expression along with a significant reduction of the resveratrol content. Further analysis showed that VvWRKY8 neither binds nor activates the promoter of *VvSTS15/21* and *VvMYB14* genes, rather it interacts directly with VvMYB14 at the N-terminus, thereby inhibiting the binding of VvMYB14 to the *VvSTS15/21* promoter. Moreover, exogenous application of resveratrol in cell suspension cultures significantly increased *VvWRKY8* expression, whereas *VvSTS15/21* and *VvMYB14* expression decreased. However, *VvWRKY8* overexpressing cells display a higher VvWRKY8 accumulation when they are treated with a proteasome inhibitor, suggesting a possible role of the ubiquitin ligase system in regulating the activity of VvWRKY8. This in turn allows the fine tuning of resveratrol biosynthesis. Together these results indicate the existence of a negative regulatory loop involving the VvMYB14 activator TF and its negative regulator VvWRKY8, the key enzymes VvSTS15/21 and the final resveratrol product that allows fine regulation of the resveratrol biosynthetic pathway. This study provides the description of a key step that deepens our understanding of the regulatory mechanisms of resveratrol biosynthesis and more generally the biosynthesis of phytoalexins in plants.

## Perspectives

New ways of investigation arise from the findings of Jiang *et al.* (2019). The fact that resveratrol production may result in the up-regulation of *VvWRKY8*, which in turn negatively regulates *VvSTS* genes to fine-tune the accumulation of this phytoalexin in grapevine tissues, triggers the question of the mechanisms underlying resveratrol interaction with VvWRKY8. As already noted, TFs are mainly located in the nucleus though some with cytosolic localization have been reported (Shang *et al.*, 2010). It could then be hypothesized that a putative nuclear resveratrol-binding receptor is activated at a high resveratrol content level and then induces *VvWRKY8* expression to negatively regulate the expression of *STS* through VvMYB14 (Box 1). Resveratrol has indeed already been found to bind to and activate a vitamin D nuclear receptor in various human tissues (Dampf Stone *et al.*, 2015). In that sense, further research is needed to explore a possible potentiation of VvWRKY8 through nuclear receptor signalling.

The existence of a negative feedback in resveratrol biosynthesis including the regulatory loop resveratrol–putative nuclear receptor–VvWRKY8–VvMYB14–VvSTS is of great importance and may have interesting applications in the molecular engineering of phytoalexin pathways in plants. In fact, the main problems encountered in this research area stem from the impossibility of obtaining ectopic production of a given phytoalexin in engineered plants (Delaunois *et al.*, 2009; Jeandet *et al.*, 2017). The study of Jiang *et al*. (2019) suggests for the first time the existence of TF negatively-regulated phytoalexin production in grapevine. It may partly explain why the overexpression of phytoalexin genes in plants does not always result in high phytoalexin production levels. The down-regulation of *VvWRKY8* expression by CRISPR-Cas technology combined with *STS* overexpression might represent an appropriate approach to improve resveratrol production and plant disease resistance. Strengthening VvWRKY8 channelling to the ubiquitin ligase proteasome pathway (Box 1) to decrease VvMYB14 repression would also constitute an interesting option for increasing resveratrol phytoalexin production in plants.
